# From Ontology to Semantic Similarity: Calculation of Ontology-Based Semantic Similarity

**DOI:** 10.1155/2013/793091

**Published:** 2013-02-28

**Authors:** Mingxin Gan, Xue Dou, Rui Jiang

**Affiliations:** ^1^Dongling School of Economics and Management, University of Science and Technology Beijing, Beijing 100083, China; ^2^Department of Automation, Tsinghua University, Beijing 100084, China

## Abstract

Advances in high-throughput experimental techniques in the past decade have enabled the explosive increase of omics data, while effective organization, interpretation, and exchange of these data require standard and controlled vocabularies in the domain of biological and biomedical studies. Ontologies, as abstract description systems for domain-specific knowledge composition, hence receive more and more attention in computational biology and bioinformatics. Particularly, many applications relying on domain ontologies require quantitative measures of relationships between terms in the ontologies, making it indispensable to develop computational methods for the derivation of ontology-based semantic similarity between terms. Nevertheless, with a variety of methods available, how to choose a suitable method for a specific application becomes a problem. With this understanding, we review a majority of existing methods that rely on ontologies to calculate semantic similarity between terms. We classify existing methods into five categories: methods based on semantic distance, methods based on information content, methods based on properties of terms, methods based on ontology hierarchy, and hybrid methods. We summarize characteristics of each category, with emphasis on basic notions, advantages and disadvantages of these methods. Further, we extend our review to software tools implementing these methods and applications using these methods.

## 1. Introduction

Recent technical innovation in high-throughput experiments has been successfully bringing about a revolution in modern biological and biomedical studies. With microarrays, expression levels of thousands of genes can be simultaneously measured [1]. With yeast two-hybrid assays, pairwise interactions between thousands of proteins can be systematically detected [2, 3]. With tandem mass spectrometry, a large number of proteins can be sequenced and characterized rapidly [4]. Indeed, high-throughput experimental techniques have enabled the collection of a vast volume of omics data, while how to organize, interpret, and use these data has now become a serious issue [5]. Each type of data explains the biological system under investigation from a specific point of view. In order to get full understanding of the system, however, one needs to integrate multiple types of data—typically coming from different laboratories and obtained using different experimental techniques. Consequently, the data should be organized in such a way that is standard across different techniques and interpretable across different laboratories. In other words, information and knowledge included in the data should be described using a set of controlled vocabulary that is standardized. Fortunately, an ontology provides us with such a standard means of organizing information [5]. 

An ontology is an abstract description system for knowledge composition in a certain domain [6]. By organizing concepts (terms) in a domain in a hierarchical way and describing relationships between terms using a small number of relational descriptors, an ontology supplies a standardized vocabulary for representing entities in the domain [7]. Particularly, in biological and biomedical domains, there have been quite a few ontologies available [5]. For example, the gene ontology (GO), including three separate domains (biological process, molecular function, and cellular component), has been widely used as a standard vocabulary for annotating functions of genes and their products across different species [8]. The human phenotype ontology (HPO) has been explored to facilitate the description of human disease phenotypes with a set of standard terms [9]. The plant ontology (PO) has been utilized to describe plant structures and growth stages [10]. Particularly, in order to achieve the goal of providing standard annotations of multiple heterogeneous data sources using common controlled vocabularies, The open biological and biomedical ontologies (OBO) Foundry has been proposed to coordinate the development of ontologies in different biological and biomedical domains [5]. Up to October 20, 2012, there have been 8 mature ontologies and 107 candidate ontologies included in the OBO Foundry, covering 25 domains, including anatomy, health, phenotype, environment, and many others [5].

Many applications using domain ontologies need to quantify the relationship between two terms [11, 12]. A suitable measure of such relationship is the semantic similarity between the terms, given the underlying domain ontology [13]. Considering the hierarchical structure of an ontology [6], the semantic similarity between two terms is in general defined as a function of distance between the terms in a graph corresponding to the hierarchical structure of the underlying ontology. However, the concrete form of the function may be refined with further knowledge about the ontology or even entities that are already annotated by using the ontology, yielding a wide variety of approaches for calculating semantic similarities of terms [14–19]. More specifically, we classify these approaches into five categories: (1) methods based on semantic distance between terms, (2) methods based on information contents of terms, (3) methods based on features of terms, (4) methods based on the hierarchical structure of an ontology, and (5) hybrid methods. Since each category of methods has its own traits, it is indispensable to know which method is suitable for the application of interest. Motivated by this consideration, we summarize characteristics of each category of methods in this paper, provide a brief review of available software implementation of these methods, and introduce typical biological and biomedical applications that rely on ontologies.

## 2. Biological and Biomedical Ontologies

The rapid development of high-throughput biological experimental techniques has enabled the explosive increase of a wide variety of omics data, while the integrated use of these data appeals for the standard annotation of multiple heterogeneous data sources using common controlled vocabularies. To achieve this goal and coordinate the development of ontologies in different domains, the open biological and biomedical ontologies (OBO) Foundry has been proposed [5]. The OBO Foundry is a collaborative experiment that aims at creating controlled vocabularies for shared use across different biological and medical domains. Participants of the OBO Foundry have agreed in advance on the adoption of a set of principles that specify the best practices for the development of ontologies, for the purpose of developing a set of interoperable humanly validated reference ontologies for all major domains of biomedical research. As shown in Table 1, up to October 20, 2012, there have been 8 mature ontologies and 107 candidate ontologies included in the OBO Foundry. These ontologies can further be classified into 25 domains, including anatomy, health, phenotype, and environment.

The 8 mature ontologies are listed in Table 2. Biological process, cellular component, and molecular function belong to the gene ontology (GO), which aims at standardizing representation of characteristics of genes and gene products across species via providing a controlled vocabulary of terms for describing annotations of gene products [20]. Specifically, biological process describes operations or sets of molecular events with a defined beginning and end. Molecular function describes elemental activities of gene products at the molecular level. The cellular component describes parts of a cell or its extracellular environment. The chemical entities of biological interest (ChEBI) provide a controlled vocabulary mainly for describing small chemical compounds, which are either products of nature or synthetic products used to intervene in the processes of living organisms [21]. The phenotypic quality (PATO) can be used in conjunction with phenotype annotations provided by other ontologies to describe qualities (such as red, ectopic, high temperature, fused, small, and edematous) for phenotypes [5, 22]. The protein ontology (PRO) is used to describe protein-related entities such as specific modified forms, orthologous isoforms, and protein complexes [23]. This ontology is separated into three domains: proteins based on evolutionary relatedness, protein forms produced from a given gene locus, and protein-containing complexes. The Xenopus anatomy and development (XAO) is designed to describe annotations of the model organism African clawed frog (*Xenopus laevis*) [24]. In this ontology, the lineage of tissues and the timing of their development are organized in a graphical view, hence facilitating the annotation of gene expression patterns, mutants, and morphant phenotypes of Xenopus. Similarly, the Zebrafish anatomy and development (XAO) provides a controlled vocabulary for annotating the anatomy of the model organism Zebrafish (*Danio rerio*) [25].

Many of the candidate ontologies have also been widely used in a variety of research areas. For example, in medical research, the human phenotype ontology (HPO) provides a means of describing phenotypic abnormalities encountered in human diseases [9]. This ontology is developed based on the Online Mendelian Inheritance in Man (OMIM) database [26] and medical literature, currently containing more than 10 thousand terms and over 50 thousand annotations to human-inherited diseases. In environmental science, the environment ontology (EnvO) is designed to support annotations of organisms or biological samples with environment descriptions [5].

## 3. Derivation of Semantic Similarity between Terms in an Ontology

### 3.1. Hierarchical Structure of an Ontology

Typically, an ontology is represented as a directed acyclic graph (DAG), in which nodes correspond to terms and edges represent relationships between the terms. In some ontologies, there is only one relationship between nodes, while in more general case, there exist more than one relationship between nodes. For example, the gene ontology defines 5 relationships between nodes: is_a, part_of, regulates, negatively_regulates, and positively_regulates [8], while the OBO relational ontology defines 13 relationships between nodes: is_a, part_of, integral_part_of, proper_part_of, located_in, contained_in, adjacent_to, transformation_of, derives_from, preceded_by, has_participant, has_agent, and instance_of [5]. 

In the DAG corresponding to an ontology, there is a node specified as the root. For every node in the ontology, there exists at least one path pointing from the root to the node. Every node in such a path is called an ancestor of the node, and the ancestor that immediately precedes the node in the path is called the parent of the node. Inversely, if a node is a parent of another node, the node is called a child of the parent. There might be more than one path from the root to a node. Consequently, a node may have several parent nodes, and vice versa. Given two nodes in an ontology, they must share a set of common ancestor nodes, and the one represents the most concrete concept is typically referred to as the lowest common ancestor of the two nodes. Discarding the direction of the edges in an ontology, there exists at least one path between every pair of two nodes. 

### 3.2. Methods Based on Semantic Distance between Terms

Given a pair of two terms, *c*_1_ and *c*_2_, a well-known method with intuitive explicitness for assessing their similarity is to calculate the distance between the nodes corresponding to these terms in an ontology hierarchy; the shorter the distance, the higher the similarity. In the case that multiple paths between the nodes exist, the shortest or the average distance of all paths may be used. This approach is commonly referred to as the semantic distance method, since it typically yields a measure of the distance between two terms. The distance can then be easily converted into a similarity measure. Four main factors are normally considered in distance-based methods as follows

1. density in the ontology graph: the higher the density, the nearer the distance between nodes;
2. depths of nodes: the deeper the nodes located in, the more obvious the difference between the nodes;
3. types of links: the normal type is is-a relation, and other relations such as part-of and substance-of are associated with the weight for edges;
4. weights of links: edges connecting a certain node with all its child nodes can vary among different semantic weights.

In the last two decades, many efforts have been devoted to building various models to measure such distance in calculating similarities. Some representative algorithms include shortest path [27], connection weight [28], and Wu and Palmer [29]. 

Rada et al. proposed the shortest path method to calculate semantic similarity based on the ontology hierarchy, suggesting that the shortest path between two nodes was the simplest approach for measuring distance between two terms [27]. In mathematics, the formula for the distance between two nodes by the shortest path was denoted by Sim(*c*_1_, *c*_2_) = 2MAX − *L*, where *c*_1_ and *c*_2_ were the compared nodes, MAX the maximum path on the hierarchy, and *L* the shortest path. The main advantage of this method was its low complexity in calculation. Rada et al. hypothesized that when only the *is-a* relationship existed in a semantic network, semantic relatedness and semantic distance were equivalent. However, this method was short of consideration for different kinds of edges as well as the semantic relatedness representing these edges.

Sussna proposed an edge weight determination scheme, which considered the first three factors: the density of the graph, depths of nodes, and types of connections [28]. In their method, the distance or weight of the edge between adjacent nodes *c*_1_ and *c*_2_ was defined as

(1) wt(c1,c2)=wt(c1→rc2)+wt(c2→r′c1)2d,given  wt(x→ry)=max⁡r−max⁡r−min⁡rnr(x),

where →_*r*_ was a relation of type *r*, →_*r*′_ its inverse, *d* the depth of the deeper node, max⁡_*r*_ and min⁡_*r*_ the maximum and minimum weights for a relation of type *r*, respectively, and *n*_*r*_(*x*) the number of relations of type *r* leaving node *x*. This method exhibited an improvement in reducing the ambiguousness of multiple sense words by discovering the combination of senses from a set of common terms that minimizes total pairwise distance between senses. However, depth factor scaling and restricting the type of a link to a strictly hierarchical relation apparently impaired the performance of the method.

Alternatively, the common path technique calculated the similarity directly by the length of the path from the lowest common ancestor of the two terms to the root node [29]. In detail, Wu and Palmer [29] took into account the position relation of *c*_1_, *c*_2_ to their nearest common ancestor *c* to calculate similarity. Here, *c* was the node with fewest *is-a *relationship as their ancestor node which appeared at the lowest position on the ontology hierarchy. In mathematics, the formula calculating similarity between *c*_1_ and *c*_2_ was denoted as

(2) $$\begin{array}{c} \text{Sim}\left(c_{1},c_{2}\right)=\frac{2H}{D_{1}+D_{2}+2H}, \end{array}$$

where *D*_1_ and *D*_2_ were, respectively, the shortest paths from *c*_1_ and *c*_2_ to *c*, and *H* the shortest path from *c* to the root. However, the calculation of similarity only cumulated shortest paths together with the consideration that all the edges were of the same weight. Hence, it might also potentially lose information of semantics represented by various types of edges existing in the ontology hierarchy.

However, in practical application, terms at the same depth do not necessarily have the same specificity, and edges at the same level do not necessarily represent the same semantic distance, and thus the issues caused by the aforementioned assumptions are not solved by those strategies [13]. Moreover, although distance is used to identify the semantic neighborhood of entity classes within their own ontologies, the similarity measure between neighborhoods is not defined based on such a distance measure.

### 3.3. Methods Based on Information Contents of Terms

A method based on information content typically determines the semantic similarity between two terms based on the information content (IC) of their lowest common ancestor (LCA) node. The information content (IC) gives a measure of how specific and informative a term is. The IC of a term *c* can be quantified as the negative log likelihood IC(*c*) = −log⁡*P*(*c*), where *P*(*c*) is the probability of occurrence of *c* in a specific corpus (such as the UniProt Knowledgebase). Alternatively, the IC can be also computed from the number of children a term has in the ontology hierarchical structure [30], although this approach is less commonly used. On the ontology hierarchy, the occurrence probability of a node decreases when the layer of the node goes deeper, and hence the IC of the node increases. Therefore, the lower a node in the hierarchy, the greater its IC. There have been quite a few methods belonging to this category. For instance, Resnik put forward a first method that is based on information content and tested the method on WordNet [18]. Lin proposed a theoretic definition of semantic similarity using information content [15]. Jiang and Conrath improved the method of Resnik by introducing weights to edges [14]. Schlicker et al. proposed a method that is applicable to the gene ontology [31]. As mentioned by Wang et al. [32], methods based on information content may be inaccurate due to shallow annotations. Lee et al. also pointed out this drawback [33].

Resnik [18] used a taxonomy with multiple inheritance as the representational model and proposed a semantic similarity measure of terms based on the notion of information content. By analogy to information theory, this method defined the information content of a term as the negative algorithm of the probability of its occurrence and the similarity between two terms *c*_1_ and *c*_2_ as the maximal information content of all terms subsuming both *c*_1_ and *c*_2_, calculated by

(3) $$\begin{array}{c} \text{Sim}\left(c_{1},c_{2}\right)=\underset{c\in S(c_{1},c_{2})}{\max}\left[-\log P\left(c\right)\right], \end{array}$$

where *S*(*c*_1_, *c*_2_) was the set of all the parents for both *c*_1_ and *c*_2_. Since the lowest common ancestor (LCA) had the maximum value of information content, recognizing the LCA of both *c*_1_ and *c*_2_ can be supported by this measure. The information content-based similarity measure was symmetric and transitive. Obvious advantages of this method were its simple calculation and easy formulation. However, in contrast to distance by Rada et al., the minimality axiom did not hold for Resnik's similarity measure. The similarity between a term and itself was the negative logarithm of its information content. Only the single term on top of the hierarchy reached the self-similarity of one. In addition, this method was only suitable for the ontology hierarchy with single relations; for example, all edges connecting terms represent only the same relationship, so it cannot be applied to the terms with either part-of relations or inferior relations.

Lin [15] proposed an alternative information theoretic approach. This method took into account not only the parent commonality of two query terms, but also the information content associated with the query terms. Three basic assumptions were normally given by Lin [15] in calculating the similarity between two terms as follows.

1. The similarity between two terms was associated with their common properties: the more the common properties, the higher their similarity.
2. The similarity between two terms was associated with their difference: the more the difference, the lower their similarity.
3. The similarity between two terms reached the maximum value when they were totally the same.

Based on the above assumptions, given terms, *c*_*i*_ and *c*_*j*_, their similarity was defined as

(4) $$\begin{array}{c} \text{Sim}\left(c_{i},c_{j}\right)=\frac{2\log P\left(c_{0}\right)}{\log P\left(c_{i}\right)+\log P\left(c_{j}\right)}, \end{array}$$

where *c*_0_ was the lowest common ancestor (LCA) of *c*_*i*_ and *c*_*j*_, and *P*(*c*_*i*_) and *P*(*c*_*j*_) were the probabilities of occurrence. Not only the information content of LCA was considered in the calculation, but also their information content was taken into account in Lin's method. This measure could be seen as a normalized version of the Resniks method. Lin's values also increased in relation to the degree of similarity shown by two terms and decreased with their difference. However, the consideration of information content of two terms themselves caused a strong dependence on the high precision of the annotation information. Consequently, exact result can be generated only when mapping relationships between compared terms and other terms in the ontology hierarchy were precisely described, while the result would be near to 0 when annotations were abstract, yielding the problem of shallow semantic annotations. In fact, the difference between two terms with abstract annotations could be large, so it might be misleading to produce similarity values according to Lin's method.

Jiang and Conrath [14] proposed a combined approach that inherited the edge-based approach of the edge counting scheme, which was then enhanced by the node-based approach of the information content calculation. The factors of depths of nodes, the density around nodes, and the type of connections were taken into account in this measure. The simplified version of the measure was given as

(5) $$\begin{array}{c} \text{Dist}\left(w_{1},w_{2}\right)=\text{IC}\left(c_{1}\right)+\text{IC}\left(c_{2}\right)-2\times \text{IC}\left(\text{LCA}\left(c_{1},c_{2}\right)\right). \end{array}$$

However, being relative measures, both the method of Lin and that of Jiang and Conrath were proportional to the IC differences between the terms and their common ancestor, independently of the absolute IC of the ancestor. To overcome this limitation, Schlicker et al. [31] proposed the relevance similarity measure. This method was based on Lin's measure but used the probability of annotation of the most informative common ancestor (MICA) as a weighting factor to provide graph placement as follows:

(6) $$\begin{array}{c} \text{Sim}\left(c_{1},c_{2}\right)=\underset{c\in S(c_{1},c_{2})}{\max}\left(\frac{2\times \log p\left(c\right)}{\log p\left(c_{1}\right)+\log p\left(c_{2}\right)}\times \left(1-p\left(c\right)\right)\right). \end{array}$$

All these measures overlooked the fact that a term can have several disjoint common ancestors (DCAs). To overcome this limitation, Couto et al. [34] proposed the GraSM method, in which the IC of the MICA was replaced by the average IC of all DCA. Bodenreider et al. [35] developed a node-based measure that also used annotation data but did not rely on information theory. Focusing on the gene ontology, their method represented each term as a vector of all gene products annotated with the term and measured similarity between two terms by calculating the scalar product of their vectors. Riensche et al. used coannotation data to map terms between different GO categories and calculated a weighting factor, which could then be applied to a standard node-based semantic similarity measure [36].

### 3.4. Methods Based on Features of Terms

In feature-matching methods, terms are represented as collections of features, and elementary set operations are applied to estimate semantic similarities between terms. A feature-matching model in general consists of three components: distinct features of term *A* to term *B*, distinct features of term *B* to term *A*, and common features of terms *A* and *B*.

Using set theory, Tversky [37] defined a similarity measure according to a matching process, which generated a similarity value based on not only common but also distinct features of terms. This approach was in agreement with an information-theoretic definition of similarity [15]. Unlike the above-mentioned models based on semantic distance [27–29], this feature-matching model was not forced to satisfy metric properties. A similarity measure based on the normalization of Tversky's model and the set-theory functions of intersection (*D*_1_∩*D*_2_) and difference (*D*_1_/*D*_2_) was given as

(7) Sim(c1,c2)=|D1+D2||D1∩D2|+μ|D1/D2|+(μ−1)|D2/D1|′,                for  0≤μ≤1,

where *D*_1_ and *D*_2_ corresponded to description sets of *c*_1_ and *c*_2_, | | the cardinality of a set, and *μ* a function that defines the relative importance of the noncommon features. The first term of a comparison (i.e., *c*_1_) was referred to as the target, while the second term (i.e., *c*_2_) was defined as the base. Particularly, intersections or subtractions of feature sets were based only on entire feature matches. This feature model allowed for representing ordinal and cardinal features, but the similarity measure did not account for their ordering. 

In addition, the Matching-Distance Similarity Measure (MDSM) by Rodríguez et al. [38] and Rodríquez and Egenhofer [7, 39] was another feature model developed for similarity measurement of geospatial terms. This category of models was based on the ratio model that extends the original feature model by introducing different types of features and applying them to terms.

### 3.5. Methods Based on Hierarchical Structure of an Ontology

Typically, an ontology is represented as a directed acyclic graph (DAG), in which nodes correspond to terms, and edges represent relationships between the terms. A parent node may have several child nodes while a child node may have several parent nodes. Some nodes have high density around them while some have low density in the hierarchy. A method based on the structure of an ontology typically uses a distance measure to quantify the similarity between two nodes in the corresponding DAG of the ontology and then uses this measure to assess the relatedness between the corresponding terms in the ontology.

There have been quite a few methods that belong to this category. For example, Rada et al. converted the shortest path length between two terms into their semantic similarity [27]. Wu and Palmer calculated the distance from the root to the lowest common ancestor (LCA) node of two terms as their semantic similarity [29]. Leacock and Chodorow calculated the number of nodes in the shortest path between two terms and then used the number with the maximum depth of an ontology to quantify the relatedness of the terms [40]. Al-Mubaid and Nguyen quantified the commonality of two terms as their similarity [41]. Wang et al. proposed to aggregate contributions of common ancestor terms to semantic values of two terms in the calculation of their semantic similarity [19]. Zhang et al. improved the method of Wang et al. and proposed the combined use of the shortest path length and the depth of the LCA node [42]. The strategies that these methods employed included lengths of shortest paths, depths of nodes, commonalities between terms, semantic contributions of ancestor terms, and many others. Although the use of these strategies has enabled the successful application of these methods to a variety of problems, the existence of a drawback in these methods is also obvious. It is common that a term in an ontology has more than one parent node in the corresponding DAG, and thus two terms may have two or more LCA nodes. However, none of the above methods take such a situation of multiple LCA nodes into consideration in their calculation of semantic similarity.

Wang et al. evaluated measures proposed by Jiang and Conrath, Lin, and Resnik and tested these measures against gene coexpression data using linear correlation [19]. They pointed out that the distance from a term to the closest common ancestor might fail in accurately representing the semantic difference between two GO terms, since two terms near to the root of the ontology and sharing the same parent should have larger semantic difference than those far away from the root and having the same parent. In addition, considering that a GO term may have multiple parent terms with different semantic relationships, they also suggested that measuring the semantic similarity between two GO terms based only on the number of common ancestor terms might fail in recognizing semantic contributions of the ancestor terms to the two specific terms. In addition, from human perspectives, an ancestor term far away from a descendant term in the GO graph should contribute less to the semantics of the descendant term, while an ancestor term closer to a descendant term in the GO graph should contribute more. 

According to the above understanding, Wang et al. presented GO as directed acyclic graphs (DAGs) in which terms form nodes and two kinds of semantic relations is-a and part-of form edges. They further defined the contribution of a GO term *t* to the semantics of GO term *A* as the *S*-value of GO term *t* related to term *A*. Formally, a GO term *A* was defined as a graph DAG_*A*_ = (*A*, *T*_*A*_, *E*_*A*_), where *T*_*A*_ was the set of GO terms in DAG_*A*_, including *A* and all of its ancestors in the GO graph, and *E*_*A*_ was the set of edges connecting GO terms in DAG_*A*_. For any term *t* in DAG_*A*_ = (*A*, *T*_*A*_, *E*_*A*_), the *S*-value related to term *A*, *S*_*A*_(*t*) was then defined as

(8) SA(A)=1,SA(t)=max⁡{we×SA(t′)t′∈children  of  (t)} (t≠A),

where *w*_*e*_ was the semantic contribution factor for edge *e* ∈ *E*_*A*_ that links term *t* and its child term *t*′. Given DAG_*A*_ = (*A*, *T*_*A*_, *E*_*A*_) and DAG_*B*_ = (*A*, *T*_*B*_, *E*_*B*_), for terms *A* and *B*, respectively, the semantic similarity between these two terms, *S*_GO_(*A*, *B*), was defined as

(9) $$\begin{array}{c} S_{\text{GO}}\left(A,B\right)=\frac{\sum_{t\in T_{A}\cap T_{B}}\left(S_{A}\left(t\right)+S_{B}\left(t\right)\right)}{\text{SV}\left(A\right)+\text{SV}\left(B\right)}, \end{array}$$

where *S*_*A*_(*t*) and *S*_*B*_(*t*) are *S*-values of term *t* related to terms *A* and *B*, respectively, and SV(*A*) and SV(*B*), defined as SV(*A*) = ∑_*t*∈*T*_*A*__ *S*_*A*_(*t*) and SV(*B*) = ∑_*t*∈*T*_*B*__ *S*_*B*_(*t*), were semantic values of terms *A* and *B*, respectively. Wang et al. further compared their measure against Resnik's method by clustering gene pairs according to their semantic similarity and showed that their measure produced more reasonable results. However, in Wang's method, the weights of the *is-a* and the *part-of *relations were empirically determined as 0.8 and 0.6, respectively, without theoretical analysis. Moreover, this method did not take into account the factor of the amount of nodes. In a subsequent study, Zhang et al. [42] pointed out that Wang's method overlooked the depth of the GO terms and proposed a measure to overcome this limitation.

Schickel-Zuber and Faltings [43] defined a similarity measure for hierarchical ontologies called Ontology-Structure-based Similarity (OSS). They pointed out that a quantitative measure of similarity should represent the ratio of numerical scores that may be assigned to each term, and thus the score of a term should be defined as a real-valued function normalized to the range of [0, 1] and should satisfy three assumptions. First, similarity scores depended on features of the terms. Second, each feature contributed independently to a score. Third, unknown and disliked features made no contribution to a score. In detail, the OSS measure first inferred the score of the term *b* from *a*, *S*(*b* | *a*), by assigning terms in the ontology an a-priori score (APS) and computing relationships between scores assigned to different terms. Then, this method computed how much had been transferred between the two terms, *T*(*a*, *b*). Finally, this method transformed the score into a distance value *D*(*a*, *b*). Mathematically, the *a-priori score* of a term *c* with *n* descendants was calculated as

(10) $$\begin{array}{c} \text{APS}\left(c\right)=\frac{1}{n+2}, \end{array}$$

implying that leaves of an ontology have an APS equal to 1/2, the mean of a uniform distribution in [0, 1]. Conversely, the lowest value was found at the root. It also implied that the difference in score between terms decreased when one traveled up towards the root of the ontology, due to the increasing number of descendants. Given two terms *x* and *z* in an ontology and their lowest common ancestor *y*, the distance value was calculated as

(11) $$\begin{array}{c} D\left(x,z\right)=\frac{\log\left(1+2\beta \left(z,y\right)\right)-\log\left(\alpha \left(x,y\right)\right)}{\max D}, \end{array}$$

where *α*(*x*, *y*) was a coefficient calculated as *α*(*x*, *y*) = APS(*y*)/APS(*x*), *β*(*z*, *y*) a coefficient estimated by *β*(*z*, *y*) = APS(*z*) − APS(*y*), and max⁡*D* the longest distance between any two terms in the ontology.

Al-Mubaid and Nguyen [41] proposed a measure with common specificity and local granularity features that were combined nonlinearly in the semantic similarity measure. Compared with other measures, this method produces the highest overall correlation with human judgments in two ontologies. In mathematics, the semantic similarity between two terms was calculated as:

(12) Sem(C1,C2) =log⁡⁡((Path−1)α×(D−depth((LCS(C1,C2))))β+k),

where *α* > 0 and *β* > 0 were contribution factors of two features, Path the length of the shortest path between the two terms, *D* the maximum depth, LCS the closest common ancestor of the two terms, and *k* a constant. Compared with other measures, this measure produced the highest overall correlation results with human judgments in two ontologies.

### 3.6. Hybrid Methods

Hybrid methods usually consider several features such as attribute similarity, ontology hierarchy, information content, and the depth of the LCA node simultaneously. One of the representative methods was OSS in which a priori score was used to calculate the distance berween two terms, and then the distance was transformed into semantic similarity [43]. Another example was the method proposed by Yin and Sheng [44], which combined term similarity and description similarity.

## 4. Derivation of Semantic Similarity of Entities Annotated with an Ontology

With the semantic similarity scores between terms in an ontology calculated using either of the above methods, the derivation of semantic similarity of entities annotated with the ontology was typically conducted using either the average rule [15] or the mean-max rule [19].

Given two sets of terms *T* and *S*, the average rule calculated the semantic similarity between the two sets as the average of semantic similarity of the terms cross the sets as

(13) $$\begin{array}{c} \text{Sim}\left(T,S\right)=\frac{1}{\left|T\right|\times \left|S\right|}\sum_{t\in T}\;\sum_{s\in S}\text{Sim}\left(s,t\right). \end{array}$$

Since an entity can be treated as a set of terms, the semantic similarity between two entities annotated with the ontology was defined as the semantic similarity between the two sets of annotations corresponding to the entities.

The mean-max rule defined the semantic similarity between a term *t* and a set of terms *T* in the ontology as the maximum similarity between the term and every term in the set as

(14) $$\begin{array}{c} \text{Sim}\left(t,T\right)=\underset{t^{\prime }\in T}{\max}\;\text{Sim}\left(t,t^{\prime }\right). \end{array}$$

Then, the semantic similarity between two sets of terms *T* and *S* was calculated as

(15) $$\begin{array}{c} \text{Sim}\left(S,T\right)=\frac{1}{\left|S\right|+\left|T\right|}\left(\sum_{s\in S}\text{Sim}\left(s,T\right)+\sum_{t\in T}\text{Sim}\left(t,S\right)\right). \end{array}$$

Finally, the semantic similarity between two entities annotated with the ontology was calculated as the semantic similarity between the two sets of annotations corresponding to the entities. 

## 5. Software for Deriving Semantic Similarity Profiles

With the above methods for calculating semantic similarity of terms in an ontology and that of entities annotated with an ontology available, a natural demand in research is the development of user-friendly software tools that implement these methods. So far, there have been quite a few such software tools available, with examples including GOSemSim [45], seGOsa [46], DOSim [47], and many others. 

Yu et al. developed GOSemSim [45] for calculating semantic similarity between GO terms, sets of GO terms, gene products, and sets of gene products. This tool was developed as a package for the statistical computing environment *R* and released under the GNU General Public License (GPL) within the Bioconductor project [48]. Consequently, GOSemSim was easy to install and simple to use. However, GOSemSim heavily depended on a number of packages provided by Bioconductor. For example, package GO.db was used by GOSemSim to obtain GO terms and relationships; packages org.Hs.eg.db, org.Rn.eg.db, org.Mm.eg.db, org.Dm.eg.db, and org.Sc.sgd.db were required in order to obtain annotations of gene products for human, rat, mouse, fly, and yeast, respectively. Although such a design scheme greatly alleviated the requirement of understanding specific formats of these annotations, the frequent access of annotation databases was typically the bottleneck of large-scale calculation of semantic similarity profiles for thousands of gene products. 

Zheng et al. proposed seGOsa [46], a user-friendly cross-platform system to support large-scale assessment of gene ontology- (GO-) driven similarity among gene products. Using information-theoretic approaches, the system exploited both topological features of the GO and statistical features of the model organism databases annotated to the GO to assess semantic similarity among gene products. Meanwhile, seGOsa offered two approaches to assessing the similarity between gene products based on the aggregation of between-term similarities. This package has been successfully applied to assess gene expression correlation patterns and to support the integration of GO-driven similarity knowledge into data clustering algorithms. This package has also assessed relationships between GO-driven similarity and other functional properties, such as gene coregulation and protein-protein interactions in *Saccharomyces cerevisiae *and *Caenorhabditis elegans*. A database consisting of semantic similarity between gene products in both *Saccharomyces cerevisiae *and *Homo sapiens *has been successfully established using seGOsa and applied to the prediction of protein interaction networks.

Li et al. developed an R-based software package (DOSim) to compute the similarity between diseases and to measure the similarity between human genes in terms of diseases [47]. DOSim incorporated an enrichment analysis function based on the disease ontology (DO) and used this function to explore the disease feature of an independent gene set. A multilayered enrichment analysis using GO and KEGG [49] annotations that helped users to explore the biological meaning implied in a newly detected gene module was also included in the DOSim package. This package has been applied to calculate relationships between 128 cancer terms, and hierarchical clustering results of these cancers have shown modular characteristics. This package has also been used to analyse relationships of 361 obesity-associated genes, and results have shown the complex pathogenesis of obesity. 

## 6. Applications of Semantic Similarity Profiles

Biological entities can be described using an ontology as a common schema as well as compared by means of semantic similarity to assess the degree of relatedness via the similarity in meaning of their annotations. In recent years, there has been a growing trend towards the adoption of ontologies to support comprehensive, large-scale functional genomics research. For example, it has been shown that incorporating knowledge represented in the gene ontology may facilitate large-scale predictive applications in functional genomics [7, 32, 50] and disease studies [12]. It has also been shown that phenotype ontologies benefit the understanding of relationship between human phenotypes [9, 11].

### 6.1. Inference of Disease Genes Based on Gene Semantic Similarity Networks

Uncovering relationships between phenotypes and genotypes is a fundamental problem in genetics. In the context of human-inherited diseases, pinpointing causative genes that are responsible for a specific type of disease will greatly benefit the prevention, diagnosis, and treatment of the disease [51]. Traditional statistical methods in this field, including family-based linkage analysis and population-based association studies, can typically locate the genetic risk to a chromosomal region that is 10–30 Mb long, containing dozens of candidate genes [52]. The inference of causative genes from these candidates hence receives more and more attention.

The inference of causative genes is typically modeled as a one-class novelty detection problem [51]. With annotations of a set of seed genes that are known to be responsible for a query disease of interest, candidate genes can be scored according to their functional similarity to the seeds and further prioritized according to their scores. To facilitate the discovery of causative genes for diseases that have no seed genes available, phenotypic similarity between diseases is incorporated. For example, [53] proposed to measure functional similarity between two genes using their proximity in a protein-protein interaction network and further designed a regression model to explain phenotypic similarity between two diseases using functional similarity between genes that were associated with the diseases. However, a protein-protein interaction network can typically cover less than half of known human genes, and thus greatly restricts the scope of application of their method.

To overcome this limitation, Jiang et al. calculated pairwise semantic similarity scores for more than 15,000 human genes based on the biological process domain of the gene ontology [12]. They demonstrated the positive correlation between semantic similarity scores and network proximity scores for pairs of proteins. Moreover, through a comprehensive analysis, they concluded that pairwise semantic similarity scores for genes responsible for the same disease were significantly higher than random selected genes. With these observations, they constructed a semantic similarity network for human genes according to a nearest neighbor rule, and they proposed a random walk model to infer causative genes for a query disease by integrating the phenotype similarity network of diseases and the semantic similarity network of human genes. They compared their methods with a number of the state-of-the-art methods and demonstrated the superior performance of their approach. 

### 6.2. Inference of Drug Indications Based on Disease Semantic Similarity Profiles

The inference of potential drug indications is a key step in drug development [11]. This problem can be defined as follows: given a query disease, a set of small chemical compounds (potential drugs) and known associations between drugs and diseases rank small molecules such that drugs more likely to be associated with the query disease appear higher in the final ranking list. Bearing an analogy to the above problem of inferring causative genes for diseases, the inference of drug indications can greatly benefit from phenotypic similarity profiles of diseases.

A typical method for the derivation of phenotypic similarity profiles of diseases is text mining. For example, van Driel et al. [54] used the anatomy (A) and the disease (C) sections of the medical subject headings vocabulary (MeSH) to extract terms from the OMIM database and further represented the OMIM record (disease) as a vector of the corresponding phenotype features. Then, they defined the similarity score between two disease phenotypes as the cosine of angle between the two corresponding feature vectors. It has been shown that such similarities are positively correlated with a number of measures of functions of genes that are known to be associated with the diseases, suggesting the effectiveness of this approach.

Recently, the availability of the human phenotype ontology (HPO) [9] provides another means of deriving the phenotypic similarity profile of diseases. Given the ontology and annotations of diseases, Gottlieb et al. [11] proposed to first calculate semantic similarity between terms in the ontology using the method of Resnik [18]. Then, treating a disease as a set of terms in the ontology, they calculated pairwise similarity between OMIM diseases. Further analysis has shown the consistent clustering of diseases according to the semantic similarity profile derived this way (Hamosh et al., 2002). With the semantic similarity profile of diseases ready, Gottlieb et al. [11] further proposed a logistic regression model to predict drug indications for diseases and showed the effectiveness of this profile.

## 7. Conclusions and Discussion

The explosive increasing of a wide variety of omics data raises the demand of standard annotations of these data using common controlled vocabularies across different experimental platforms and different laboratories. Biological and biomedical ontologies [5], as abstract description systems for knowledge composition in the domain of life sciences, provide structured and controlled representations of terms in this field and, thus, reasonably meet this end. Targeting on the problem of quantifying the relationships between terms in an ontology, and relationships of entities annotated with an ontology, we have summarized a number of existing methods that calculate either semantic similarity between terms using structures of an ontology, annotations of entities, or both. We have further extended the review to the calculation of semantic similarity between entities annotated with an ontology and summarized typical applications that made use of biological and biomedical ontologies.

Although there have been quite a few methods for calculating semantic similarity between terms in biological and biomedical ontologies, the correctness of these methods largely depends on two factors: the quality of the annotation data and the correct interpretation of the hierarchical structure of an ontology. Particularly, for methods that depend on information contents of terms, noise existing in annotation data can adversely affect the correct estimation of the information contents and further bring noise into the resulting semantic similarity. For example, in gene ontology, a large proportion of annotations is inferred electronically by sequence similarity of gene products or other annotation databases. Whether such inferred annotations should be used in the calculation of information contents or not is still an open question. Furthermore, some gene products have been studied in more detail, while knowledge about some gene products is very limited. As a result, available annotations are biased towards heavily studied gene products, and quality of annotations is also biased. Such biased in annotations will also adversely affect the correctness of the derived information contents. 

On the other hand, many biological and biomedical ontologies have multiple types of relationships between terms (e.g., is_a, part_of, etc.), and thus methods rely on structure of an ontology need to properly weigh different types of relationships between terms. How to determine such weight values, however, is an open question. For example, although Wang et al. [19] have suggested the weights of 0.6 and 0.8 for is_a and part_of relationships in gene ontology, respectively, whether these values are suitable for other ontologies is not systematically evaluated. Furthermore, for ontologies that have even more types of relationships, the determination of the weight values becomes a more serious problem.

As for applications that make use of ontologies, the problem needs to be cared about is the circularity. For example, information contents are calculated by using annotations, and thus using similarity in annotations to evaluate the goodness of semantic similarity derived from information contents is not appropriate. A direct consequence of overlooking such circularity will be the overestimation of the performance of an application—good in validation but poor in real situation.

## Figures and Tables

**Table 1:** Domains in the OBO Foundry.

Index	Domain	Number
1	Adverse events, health	1
2	Algorithms	1
3	Anatomy	39 (3)
4	Anatomy and development	1
5	Anatomy, immunology	1
6	Behavior	1
7	Biochemistry	3 (1)
8	Biological function	1 (1)
9	Biological process	3 (1)
10	Biological sequence	1
11	Environment	3
12	Experiments	8
13	Genomic	1
14	Health	12
15	Information	1
16	Lipids	1
17	Medicine	2
18	Molecular structure	1
19	Neuroscience	3
20	Phenotype	8 (1)
21	Proteins	6 (1)
22	Provenance	1
23	Resources	1
24	Taxonomy	4
25	Other	11

	Total	115 (8)

**Table 2:** Mature ontologies in OBO.

Title	Domain	Prefix
Biological process	Biological process	GO
Cellular component	Anatomy	GO
Chemical entities of biological interest	Biochemistry	CHEBI
Molecular function	Biological function	GO
Phenotypic quality	Phenotype	PATO
Protein ontology	Proteins	PR
Xenopus anatomy and development	Anatomy	XAO
Zebrafish anatomy and development	Anatomy	ZFA
